# Notice from New Treasurer

**Published:** 1842-09

**Authors:** Leonard Mackall

**Affiliations:** Treasurer Am. Soc'y of Dental Surgeons.


At the meeting of the society in July last, the undersigned was elected Treasurer. He intends making out accounts against all those who are in arrears both for the Journal and for annual dues, and he would most earnestly request the members and others who are indebted, to make immediate payment, as it will be impossible for the Treasurer to sustain the honour and credit of the society without funds.

Agents also are requested to hold themselves in readiness to meet the drafts which the Treasurer will shortly draw on them, or if any of them shall have opportunities to make remittances, they will please do so.

LEONARD MACKALL,
*Treasurer Am. Soc'y of Dental Surgeons.*

